# Accelerating Organizational Change to Build Mentorship Culture in Zambian Universities

**DOI:** 10.5334/aogh.4032

**Published:** 2023-02-20

**Authors:** Wilbroad Mutale, Selestine H. Nzala, Marie H. Martin, Elizabeth S. Rose, Douglas C. Heimburger, Fastone M. Goma

**Affiliations:** 1 University of Zambia, ZM; 2 Vanderbilt University Medical Center, US

**Keywords:** Mentorship, Culture, Organizational change, Faculty development, Low- and middle-income countries, Zambia

## Abstract

Strong cultures of mentorship and research remain underdeveloped at many African universities, threatening future knowledge generation essential for health and development on the continent. To address these challenges, a mentorship program was developed in 2018 at the University of Zambia with an aim to enhance the institutional culture of mentorship and to build institutional capacity through an innovative ‘train the trainer’ faculty development model. In this study, we documented perceptions of lived experiences related to mentorship culture by following trainers and trainees and their mentees over two years. We analyzed these perceptions to assess changes in institutional attributes regarding mentorship. We identified positive change in institutional culture towards mentorship, and this change appeared sustainable over time. However, a slight decrease in indicators for year two emphasizes the need for a continued culture of learning rather than assuming that one-off training will be sufficient to change culture.

## Introduction

Organizational change (OC) has a profound impact on an organization’s performance, innovation, creativity, and strategy as well as employee engagement and performance [1,2,3,4,5,6,7]. Well-managed OC can have a positive impact on an organization’s performance [8].

Yet institutional perspectives on change and learning recognize that institutions are not easily altered [9]. Weber [10] refers to this inhibition to change as the ‘Iron Cage of Bureaucracy,’ where bureaucracy becomes a universal and compelling iron cage that is difficult to escape. The static rule structures that exist can limit and constrain decision-making powers, allocation of resources, and new courses of action [11]. These structures are shaped by public institutional contexts that include political pressures, social expectations, legal constraints, and economic forces.

Within this context, public universities are bound by regulations, policies, and legislation that differ from the private sector. Institutional learning in this context is constrained by the inflexibility of a large and complex system, and innovation can be considered risky. Literature has shown that some African universities remain inflexible, and the culture of mentorship and research remain underdeveloped, threatening future knowledge generation that will be essential for health and development in general [12,13,14]. While there is no consensus definition of culture, it is often understood as dynamic patterns of thinking and feeling that are understood through symbols, values, and artefacts [15]. Kroeber and Kluckhohn [15] described cultural systems as products of behavior and actions that sustain cultures.

## The University of Zambia Faculty Mentor Training Program

As part of a training program to enhance the PhD programs at the University of Zambia (UNZA) [16], a needs assessment was conducted to understand institutional and individual constraints to the culture of mentorship, which was described as weak. Participants identified a lack of mentoring culture and systems, organized mentoring structures, and available trained mentors. Based on this needs assessment, the UNZA Faculty Mentor Training Program was developed in 2018 for senior and junior faculty who mentor graduate students. The program was co-developed by faculty and staff at UNZA and the Vanderbilt Institute for Global Health with an aim to build institutional capacity through an innovative ‘train the trainer’ faculty development model. In this capacity strengthening model that promotes program sustainability, UNZA senior faculty trained junior faculty to co-lead the program in subsequent years.

The program provided a structured approach to mentoring practices for senior and junior faculty to better support graduate students at UNZA. The needs assessment and a literature review of mentorship best practices informed the development of the program’s ten modules, which include topics on communication, conflict resolution, diversity, effective leadership, establishing goals, ethics, evaluating mentorship, fostering independence, professional responsibilities, and writing one’s mentorship philosophy. The modules are taught over five day-long workshops. In preparation for workshops, trainees read peer-reviewed articles on mentorship topics. During the workshop, they engage in small and large group discussions and analyze case studies on those topics. Trainees also craft their own mentorship philosophy to guide their practice. Since the program launch in 2018, 71 faculty members have been trained through this program, which has been replicated with a goal to train all health sciences faculty at UNZA.

## Methods

We conducted a program assessment using a standard ‘mentoring culture audit’ developed by Zachary [17]. The audit, which consists of 50 best practice items, is designed to help diagnose, analyze, and prioritize organizational focus in improving mentoring culture [17]. Our aim was to understand the extent to which UNZA mentorship culture changed over time. Fifteen items (indicators) were selected as areas of focus to be included this assessment. The assessment was sent electronically using Research Electronic Data Capture (REDCap) [18,19], a secure web platform for database management, to program participants in the first cohort (facilitators and faculty participants, n =21) and their mentees (n = 78), at the end of the program (i.e., baseline), and at 1 year and 2 years post training. Respondents rated each item on a Likert scale from one (strongly disagree) to seven (strongly agree). The study protocol and evaluation instruments were approved by the University of Zambia Biomedical Research Ethics Committee (#IRB00001131 of IORG000774) and the Vanderbilt University Institutional Review Board (#180703).

### Results

Table 1 shows trends and changes in the 15 indicators. We noted an overall mean increase from baseline to year 2, with the maximum increase noted one year after completing the program. Of note, after exposure to the course, organization learning was actively promoted, and organizational culture was more supportive of mentorship. There were also benefits that accrued to leadership. These results were true for both program participants and their mentees.

**Table 1:** **Participants’ and Mentees’ mean ratings of mentorship culture indicators.** (1 = strongly disagree; 7 = strongly agree).


INDICATORS	PARTICIPANTS’ RATINGS (MEAN)			MENTEES’ RATINGS (MEAN)		
	
	BASELINE	1 YEAR POST-PROGRAM	2 YEARS POST-PROGRAM	BASELINE	1 YEAR POST-PROGRAM	2 YEARS POST-PROGRAM

Organizational leaders actively promote individual and organizational learning	4.9	4.4	4.8	4.1	4.9	4.5

The organizational culture supports mentoring	4.0	4.8	4.9	3.8	4.6	3.9

Mentoring as practiced in the organization incorporates best-practice models of adult learning	4.1	4.2	4.6	3.4	4.1	3.8

The right people are in place to support, manage, and coordinate mentoring efforts	4.4	4.5	4.4	3.9	4.6	3.8

Mentoring partners are supported in taking time for mentoring	3.9	4.0	4.1	3.9	4.3	3.5

Technology and knowledge resources that promote and support mentoring are accessible, up-to-date, and put to use	3.8	3.3	4.3	3.1	3.4	3.2

Mentoring as it is currently practiced clearly aligns with the organization’s values	3.4	3.8	4.4	3.4	4.3	3.7

Mentoring is linked to leadership development	4.3	4.4	4.4	4.2	5.5	4.9

Mentoring results are measured over time	4.1	4.0	3.1	3.3	4.4	4.2

Mentoring process improvements are timely	4.3	4.6	4.4	3.6	4.6	4.0

Excellence in mentoring is recognized, rewarded, and celebrated	3.3	3.6	3.6	2.8	4.2	3.1

People participate in mentoring relationships enthusiastically	3.8	4.7	3.6	3.2	4.0	3.3

Mentors and mentees request additional opportunities to learn how to increase their mentoring effectiveness	3.8	4.4	3.8	3.6	4.6	3.8

Individual mentoring partnerships meet regularly	4.1	4.5	3.8	3.5	4.1	3.2

Mentors and mentees make time for mentoring a priority	3.9	4.2	3.7	3.3	4.2	3.4

Overall	4.0	4.2	4.1	3.5	4.4	3.8


Overall, participants’ mean ratings of UNZA’s mentorship culture as measured by the 15 indicators increased by 3% two years post-program. The areas of greatest growth included ‘*Mentoring as it is currently practiced clearly aligns with the organization’s values*’ and ‘*The organizational culture supports mentoring*’ (increases of 29% and 22%, respectively). These areas of growth indicate that participants noticed an increase in organizational support for mentorship activities.

Mentees’ mean ratings of the mentoring culture increased by 6% two years after the program. The largest areas of growth were in the indicators ‘*Mentoring results are measured over time*,’ ‘*Mentoring is linked to leadership development*,’ ‘*Mentoring as practiced in the organization incorporates best-practice models of adult learning*,’ and ‘*Organizational leaders actively promote individual and organizational learning*’ (27%, 17%, 11%, and 11%, respectively). Similar to program participants, their mentees noted increased organizational support for mentorship. However, they also perceived increases in organizational support for leadership and learning. It appears that mentorship training and growth in the mentorship culture potentially had spillover effects to leadership and learning.

## Implications

Institutional mentorship culture is important for developing the next generation of researchers, scientists, and practitioners. While mentorship training programs exist globally, their impact on institutional culture beyond individual benefits has not been well described. In this study, we documented baseline culture of mentorship and institutional leadership and followed participants and their mentees over two years to assess changes in institutional attributes that support mentorship. Cultural change often occurs slowly over a long time period, and seemingly small shifts can be impactful in an organization. Although small, we identified positive changes in institutional culture towards mentorship, and this change appeared sustainable over time. The slight dip noted in year 2 indicators emphasizes the need for a continued culture of learning rather than assuming one-off training will be sufficient to change the culture. Incentives coupled with refresher courses and systems that reward mentorship will be needed to help continue cultural change.

Two core principles guided this capacity strengthening approach. First, senior UNZA faculty directed and led the curriculum development process in consultation with Vanderbilt partners to facilitate context-appropriateness, ensure sustainability, enhance their knowledge, and hone their mentoring skills. Second, junior faculty participated in the program, developing their mentoring skills to guide the next generation of graduate students and future faculty. Because UNZA senior faculty led the curriculum development and implemented the program, such an approach could result in institutional change and reduce inflexibility that is known to exist in such institutions. We noted that at baseline, senior faculty did not recognize the gaps in mentorship and institutional cultures that existed at UNZA. This might have hindered real change. Instead of addressing these gaps through traditional avenues such as policy briefs and institutional surveys, the approach adopted here included development and implementation of a mentorship training guided by best practices in the field. The literature supports our results that culture can change but such change requires time and deliberate investment. Boss et al. [20] reported long-term culture change occurring over thirty years that included improved organization climate and leader effectiveness spurred by an organization development program to address change in the public sector.

## Conclusion

Solving complex global challenges requires a broad cross-section of expertise in diverse fields. Formalized mentorship programs such as the UNZA Faculty Mentor Training Program help strengthen the human resource capacity needed to build the next generation of leaders, researchers, and practitioners to address local and global challenges. Such programs require buy-in and support from institutions, governments, and individuals for long-term sustainability. One of the UNZA senior mentors remarked, “I am now an advocate for structured mentorship at the school level.” This perspective speaks to the importance of viewing mentorship as an institutional value driven by both senior and junior leadership.

The positive outcomes of mentorship for both the mentor and mentee, as noted in literature and now reported in our study, have prompted the attention of major research agencies like the United States National Institutes of Health to advocate for mentorship as part of institution capacity strengthening. We support these initiatives to promote institutional culture of mentorship as a catalyst to research and leadership development for the next generation of researchers and scientists in Africa and other regions.

## Additional File

The additional file for this article can be found as follows:

10.5334/aogh.4032.s1 Trainee and mentee data set. Data and analyses from the 2018 and 2019 cohort trainees and their mentees.
